# PMMA-ITO Composite Formation via Electrostatic Assembly Method for Infra-Red Filtering

**DOI:** 10.3390/nano9060886

**Published:** 2019-06-14

**Authors:** Wai Kian Tan, Atsushi Yokoi, Go Kawamura, Atsunori Matsuda, Hiroyuki Muto

**Affiliations:** 1 Institute of Liberal Arts and Science, Toyohashi University of Technology, 441-8580 Toyohashi, Japan; yokoi@ee.tut.ac.jp; 2 Department of Electrical & Electronics Information Engineering, Toyohashi University of Technology, 441-8580 Toyohashi, Japan; gokawamura@ee.tut.ac.jp (G.K.); matsuda@ee.tut.ac.jp (A.M.)

**Keywords:** electrostatic adsorption, electrostatic assembly, composite, infra-red filtering

## Abstract

Formation of functional composite materials with desired properties is important for advanced application development. However, formation of a homogenous composite material via conventional mixing methods still remains a challenge due to agglomeration. Therefore, this work reports and demonstrates the formation of a homogeneous poly(methylmethacrylate) (PMMA)-indium tin oxide (ITO) composite with high visible light transparency (up to 90%) with an excellent shielding effect of infra-red (IR) via a facile electrostatic assembly method. This PMMA-ITO composite with good transparency and an IR shielding effect has good potential to be used in the automobile industry for vehicle windscreens as well as in heat preservation or preventive technology. The IR shielding rate is demonstrated to be controllable by changing the amount of ITO nanoparticles additive. This finding would provide a platform for development of IR optical related polymeric composite materials.

## 1. Introduction

In materials design, polymer matrix nanocomposites are deemed to be a new generation of high performance materials with desired properties. This is largely due to the controllability of the physical and chemical properties by adjusting the composition of organic and inorganic materials in the composite. It is well known that poly(methyl methacrylate) (PMMA) is a good material with potential to replace glass for many applications. The properties of PMMA such as good optical clarity, high mechanical strength, good thermal stability and amorphous polymer processing dimensional stability have made it a good host candidate for the addition of transparent functional fillers to obtain a desired functional transparent composite material [1,2,3]. On the other hand, indium tin oxide (ITO) also possesses favorable optical and electrical properties such as good transparency in the visible light region and high absorbance in the UV region, as well as exhibiting a shielding effect on infra-red (IR) light by reflectance [4,5]. The feasibility of large area ITO film fabrication has enabled its application for electronic display screen, sensors and solar cells [6,7,8]. By using physical methods such as radio frequency magnetron sputtering, Baia et al. reported on large area ITO film formation involving the processing parameter and aging effect on the electro-optical characteristic of the film [9,10]. In alignment with today’s sustainable development goals, the prevention of heat loss using IR thermal shielding technology and automotive glass will play an important role towards energy conservation [11,12,13]. However, as ITO is relatively expensive, it is not cost-effective to solely use ITO films for IR filtering [14]. Furthermore, it has been reported that bulk matrix with ITO nanoparticles can exhibit higher strength and stability than ITO films and powders [15]. Therefore, fabrication of a composite material that possesses an IR shielding property with good transparency is one of the solutions towards achieving this goal. There are several works that reported on the electrical characteristic studies of PMMA-ITO composites, which focused more on the phase segregation via percolation by changing the composition used [16,17]. The application of PMMA-ITO composites for IR filtering is yet to be investigated and therefore it is an interesting application to venture into.

In the formation of composite materials, homogenous distribution of the additive materials within the matrix is indispensable to obtain uniform and desired properties. However, conventional mixing methods available today, such as mechanical milling, are deemed insufficient to achieve a good homogenous mixture due to the occurrence of agglomeration. Hence, a new method via electrostatic assembly is used in this work in order to fabricate PMMA-ITO composites with good transparency and a controllable IR filtering effect. Chen et al. recently reported on an interesting in-situ formation of monodisperse crystalline ITO nanoparticles with a size of approximately 20~30 nm within a silica glass matrix. The tailoring of the ITO nanoparticles size and distribution was controlled by regulating the mesopores of silica glass. However, the fabrication process was time-consuming (up to 36 h) and required high temperature heat-treatment up to 1000 °C [15]. Moving towards sustainable development, a new processing technology with low environmental load and energy consumption is indispensable. Furthermore, the properties of the composite materials prepared via this method should not be compromised and should be applicable for practical application. Previously, our group has demonstrated the formation of a composite material by an electrostatic assembly method for different applications, such as optical-properties-controlled film formation at room temperature using aerosol deposition [18] and selective laser sintering [19].

The fabrication of PMMA-ITO composite material has good potential to be used as lightweight IR shielding material or IR active fillers for heat energy conservation applications, such as energy saving windows and heat-reflecting shields. In a more advanced application such as laser-holography, IR absorbing ITO nanoparticles that are distributed within a PMMA matrix would enable the adsorption of the IR laser irradiation to generate the heat required for PMMA degradation, causing a turbid appearance for 3D holographic writing inside a transparent PMMA block [20]. Nevertheless, for such precision technology, the design and distribution of ITO nanoparticles within the polymer matrix is very crucial. Furthermore, the excessive use of ITO as a transparent electrode has resulted in the rising cost of ITO day by day, and indium is facing depletion in the near future. Until the discovery of other optional transparent conductive oxide (TCO) materials as replacement, efficient utilization of ITO is important to overcome this problem [14].

## 2. Materials and Methods

The experiments were carried out using commercially available poly(methylmethacrylate) (PMMA) particles (average particle diameter 12 μm, Sekisui Chemical, Tokyo, Japan) and indium-tin oxide (ITO) nanoparticles (average particle size 50 nm, Sigma Aldrich, Tokyo, Japan). The polycation and polyanion used were polydiallyldimethyl ammoniumchloride (PDDA) (average molecular weight 100,000 to 200,000, Sigma-Aldrich) and polysodium styrenesulfonate (PSS) as polyanion (average molecular weight 70,000, Sigma-Aldrich), respectively. The surfactant used for the initial coating onto PMMA was sodium deoxycholate (SDC). After that, an alternative layer of PDDA-PSS-PDDA was coated onto PMMA to induce positive zeta-potential-modified PMMA primary particles. As for the ITO nanoparticles, the surface charge was modified using PSS to obtain a negative zeta-potential surface. Due to the nanoscale size of ITO nanoparticles, the long-chained PSS is thought to be too long and could induce agglomeration as a result of the wrapping effect of the PSS chain with a group of ITO nanoparticles. Therefore, in a subsequent study, the surface charge of ITO nanoparticles was also modified using pH adjustment and used for electrostatic assembly for comparison. The pH adjustment was carried out by aqueous HCl or NaOH titration and the change in pH and zeta potential were then measured. An ultrasonic homogenizer (QSonica, LLC., Q 700, Newtown, CT, USA) was used to disperse the agglomerated ITO nanoparticles in a solution and the zeta potential was measured using measurement equipment from Otsuka Electronics Co. Ltd. (Osaka, Japan), ELSZ-1 and Micro Tech Nission (Doha, Qatar), ZEECOM Co. Ltd. (Chiba, Japan). Then, the oppositely charged PMMA particles (positive) and ITO nanoparticles (negative) were mixed and stirred to obtain the electrostatically assembled PMMA-ITO composite particles. The suspension was then dried to obtain the composite powder. For the optical properties evaluation of the PMMA-ITO composite obtained, the composite powders were pressed into pellets using a hot press with a pressure of 200 MPa at 170 °C for 30 min. The morphological structure of the composites obtained was observed using an S-4800 Field Emission Scanning Electron Microscope (FE-SEM, Hitachi S-4800, Tokyo, Japan), while the optical properties of the nanocomposite pellets were evaluated using a UV-Vis spectrometer (JASCO International V-670, Tokyo, Japan).

## 3. Results and Discussion

**Figure 1:**
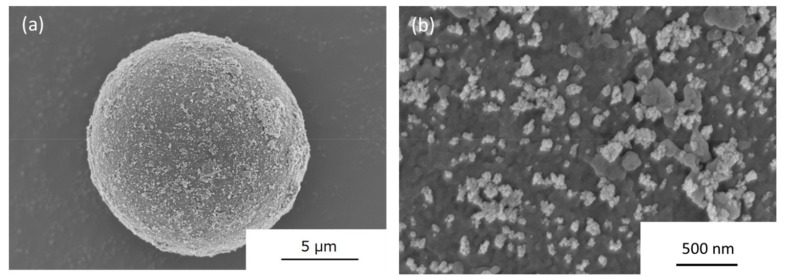
Scanning electron microscope (SEM) images of the PMMA-ITO composite obtained after electrostatic assembly adsorption of ITO nanoparticles that were surface-modified using PSS in (**a**) low magnification and (**b**) high magnification views.

The SEM images of the PMMA-ITO composite particles obtained after electrostatic assembly using ITO nanoparticles that were surface modified with PSS are shown in Figure 1. From the low magnification image in Figure 1a, a quite homogenous distribution of the ITO nanoparticles was observed. Small patches of ITO agglomeration were also observed. The low degree of agglomeration could be observed clearly upon high magnification observation as shown in Figure 1b. As PSS is a long polymeric chain and given the nanoscale size of the ITO nanoparticles at approximately 50 nm, it is thought that the long chain of PSS could have formed a random matrix chain and wrapped several ITO nanoparticles causing this phenomenon. Furthermore, the PSS-modified ITO nanoparticles also have a tendency to deposit onto the surface defects of a PMMA particle. This phenomenon was also observed by Peng et al., where PSS-modified ITO nanoparticles fabricated in their work demonstrated preferential deposition on the surface defects on the paper’s fibril structure [21]. A combination of both the abovementioned factors resulted in the formation of patches of ITO nanoparticles on the surface of PMMA particles.

**Figure 2:**
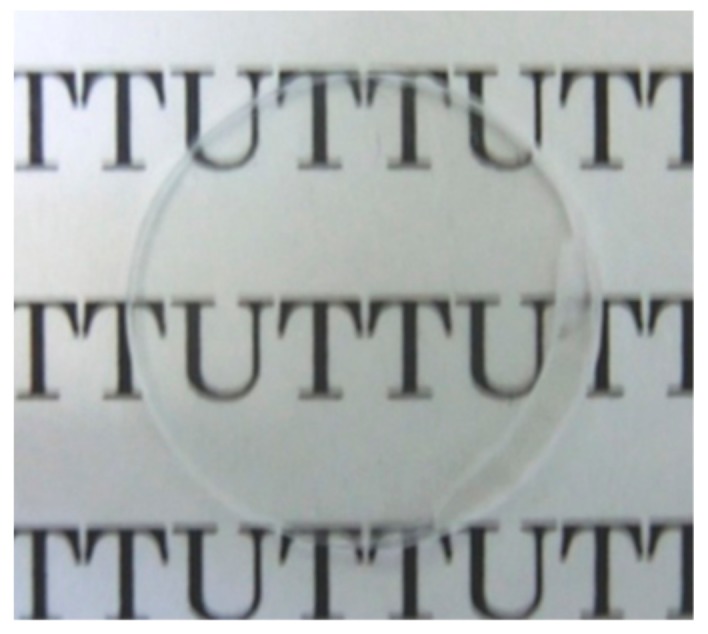
Photograph of the pellet obtained after being hot-pressed at 200 MPa at 170 °C for 30 min.

A photograph of the pellet obtained after hot-press using the PMMA-ITO composite powders as raw material is shown in Figure 2. It can be seen that the pellet demonstrated a good glass-like transparency. By using the as-received PMMA particles without any surface modification treatment, the adsorption of ITO nanoparticles on its surface was difficult due to the almost negligible surface charge and poor wettability of PMMA. Therefore, it is important to have an initial layer of surfactant, SDC on PMMA prior to subsequent electrostatic assembly of PDDA-PSS-PDDA layers to induce higher surface zeta potential for more stable adsorption of ITO nanoparticles.

**Figure 3:**
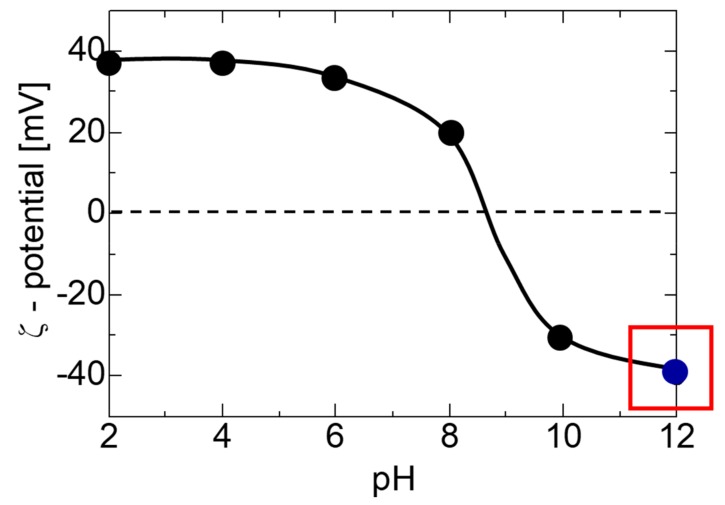
The zeta potential versus pH of an ITO nanoparticle suspension after the pH adjustment process.

As the nanoparticle size of the filler greatly influence its application in transparent polymers, even slight agglomeration or size increase would result in steep light scattering [20]. On the other hand, nanoparticles that have a high specific surface area possess a strong tendency towards agglomeration causing lower distribution homogeneity. In an attempt to further improve the transparency of the PMMA-ITO composite pellet, it is important to eliminate the slightest agglomeration generated by PSS. Therefore, the surface charge of ITO nanoparticles was modified by pH adjustment and then used for the electrostatic assembly in the subsequent investigation. The zeta potential versus pH of the ITO nanoparticles containing solution is shown in Figure 3. The zeta potential of the ITO nanoparticles suspension, which is pH dependent, can be adjusted from approximately +40 to −40 mV at pH 2 and 12, respectively. At pH 12, the zeta potential of the ITO nanoparticles obtained was −40 mV, which is sufficient to maintain the ITO particles in a colloidal condition and avoid agglomeration [21]. The pH adjusted ITO nanoparticles with strong negative surface charge would generate a strong repulsion force among the ITO nanoparticles allowing the formation of a homogenously dispersed ITO nanoparticles suspension. Upon addition of the ITO nanoparticles suspension into the solution containing positively-charged PMMA particles, the strong electrostatic attraction of ITO nanoparticles towards the PMMA particles would then lead to the electrostatic assembly of PMMA-ITO composite particles. The zeta potential-pH adjustment results obtained also corroborate with the finding reported by Peng et al. where they used pH 3 to adjust the surface charge of ITO nanoparticles to be positively charged.

**Figure 4:**
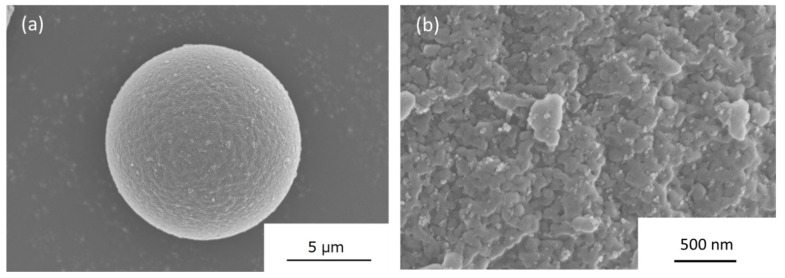
SEM images of the PMMA-ITO composite obtained after electrostatic assembly adsorption of ITO nanoparticles that were surface-modified using pH adjustment in (**a**) low magnification and (**b**) high magnification views.

The coverage percentage of ITO nanoparticles on PMMA was adjusted to be at 10 vol.% for this investigation. The morphological SEM images obtained are shown in Figure 4. From the low magnification images in Figure 4a, it is difficult to locate the ITO nanoparticles due to both the nanoscale size of the nanoparticles and the improved dispersion achieved using a pH adjustment method compared to a PSS polyelectrolyte adjustment. Upon high magnification observation as seen in Figure 4b, the ITO nanoparticles were observed to be homogeneously adsorbed onto the surface of the PMMA particle. A PMMA-ITO pellet was then fabricated using the as-obtained composite powder via the abovementioned hot-press condition. With the exceptionally low degree of ITO nanoparticle agglomeration, higher surface coalescence between the PMMA–PMMA interface was also promoted, which could lead to improved mechanical properties [16].

**Figure 5:**
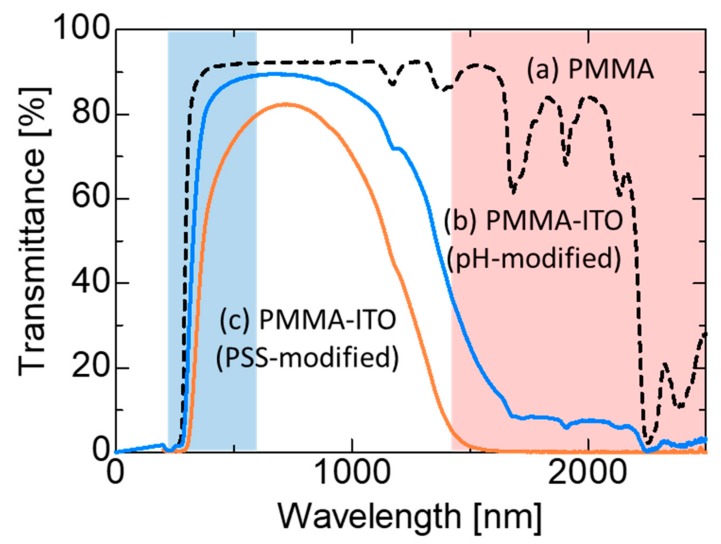
Transmittance properties of (**a**) PMMA and the PMMA-ITO composite pellets obtained using ITO nanoparticles modified using (**b**) pH adjustment and (**c**) PSS.

The optical transmittance properties of the PMMA and PMMA-ITO pellets fabricated using surface charge modification by PSS and pH adjustment are shown in Figure 5. PMMA pellets exhibited approximately 90% transmittance from a wavelength of 300 to 2200 nm. It is interesting to note that the PMMA-ITO composite pellet (PSS-surface-modified) demonstrated transmittance up to 80% at the visible region from approximately the 350 to 750 nm region and a complete IR light cut-off from 1500 nm as shown in Figure 5c. Meanwhile, as for the PMMA-ITO pellets fabricated using pH-adjusted ITO nanoparticles, the transmittance in the visible region was observed to increase to approximately 90% exhibiting a better transparency compared to those obtained using ITO nanoparticles that were surface-modified using PSS. In a work reported by Katagiri et al., ITO nanoparticles that were dispersed within a silica matrix exhibited a complete IR light shielding with wavelengths longer than 1400 nm, obtained using 10% Sn doping of ITO nanoparticles. The transmittance in the visible region reported is 80%, which is comparable with the PMMA-ITO composite fabricated using the PSS-surface-charge modification. They mentioned that the surface plasmon resonance achieved is the highest among the samples investigated in their study (between 3~30 mol% Sn doping) and generated the highest electron density [13]. In a recent publication, a similar observation was also reported by Matsui et al., where the plasmon modes contributed an important role in enhancing reflectance in the near-IR range in their study involving plasmonic responses of assembled films consisting of ITO nanoparticle films placed on different substrates [11].

Despite the higher transmittance, the sample still exhibited a very good IR light cut off beyond 1500 nm wavelength with approximately 10% of IR light transmittance. The transmittance exhibited at the visible region was one of the highest transmittance values obtained using a PMMA-ITO composite. The shielding of IR light is due to the absorbance and reflectance of an IR ray by ITO nanoparticles that were distributed within the PMMA matrix [20]. As ITO nanoparticles demonstrate IR plasmonic characteristics, the electric field interaction between ITO nanoparticles generate plasmonic coupling [12]. The electric field localized between the ITO nanoparticles is dependent on the inter-particle distance. According to Matsui et al., when the gap is less than the diameter of a nanoparticle, remarkable enhancement of the electric field is achieved [11]. Three-dimensional (3D) field interactions along the in-plane and out-plane direction would then provide high light reflections in the near- and mid-IR regions [11]. The 3D electric field coupling phenomenon would generate resonant splitting of plasmon excitations to the quadrupole and dipole modes and therefore, obtaining selective high reflections in the IR range [22]. As the control of the inter-particle distance still remains a challenge, our novel method of electrostatic assembly allows good ITO nanoparticles distribution on PMMA, which exhibit better controllability in the adjustment of ITO nanoparticles inter-particle gap.

**Figure 6:**
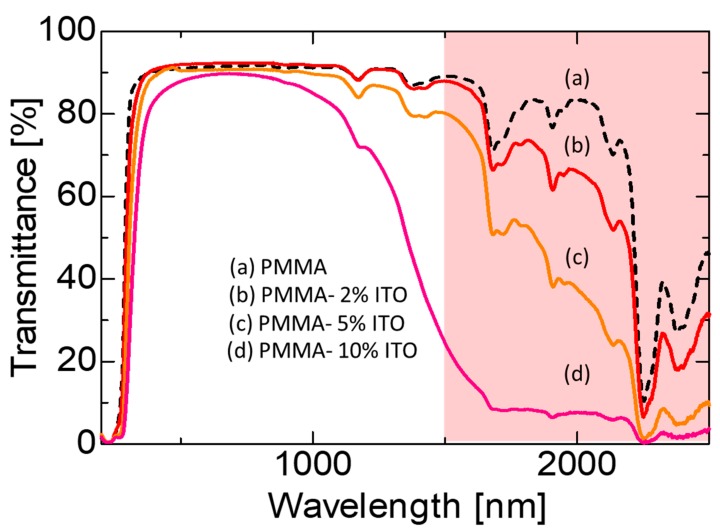
Transmittance properties of the PMMA-ITO composite pellet obtained using ITO nanoparticles modified using pH adjustment.

To further demonstrate the controllability of the IR transmittance property, the coverage of ITO nanoparticles used was varied between 2, 5 and 10 vol.% and the transmittance properties are compared in Figure 6. From the comparison, it is noteworthy that the transmittance at the visible region is almost similar at approximately 90%, while IR transmittance varied with the ITO nanoparticles coverage percentage. At the wavelength of 2000 nm, the IR transmittance is at 70%, 40% and 8% using the ITO decorated PMMA particles with a coverage percentage of 2, 5 and 10 vol.%, respectively. These findings demonstrated the controllability of the IR transmission by adjusting the amount of ITO nanoparticles that were homogenously decorated within a PMMA matrix while exhibiting an excellent visible light transmission. By controlling the amount of ITO nanoparticle distribution within the PMMA matrix in a homogenous fashion using electrostatic assembly, the control of inter-particle gap of ITO nanoparticles is feasible. Subsequently, the near 3D distribution of ITO nanoparticles in the matrix would allow the generation of electric field coupling and resonant splitting of plasmon excitations for IR rays reflectance [11,22]. In comparison with the work reported by Arlindo et al., they incorporated ITO nanowires into PMMA and the optical transmittance reduced linearly with the amount of ITO addition [3]. By varying the amount of ITO nanowires from 1 to 10 wt.%, the visible light transmittance reduced quite significantly from approximately 90% to 45%, respectively. However, they did not investigate the transmission of the IR rays of their composite. Capozzi et al. investigated the mechanism of PMMA-ITO composites and their percolation using mechanically mixed powders of both materials. Agglomeration of ITO nanoparticles was observed in their work and displacement occurred during hot-press compaction causing concentrated edges of polyhedral PMMA particles after hot-pressing [17]. The transmittance of the PMMA-ITO composites in the visible region reduced drastically from approximately 80% to 50% and 20% with the addition of 0, 0.4 and 0.99 wt.% ITO nanoparticles, respectively. In this work, a novel formation of homogeneous ITO nanoparticles distribution across PMMA surface enabled a good transmittance in the visible light-region and simultaneously, a possible controlled IR light transmission was also demonstrated. Further study on the electrical conductivity as well as the elucidation of its different formation mechanism paths will be conducted in future study. In this case, incorporation of higher volume percentage of ITO nanoparticles is required in order to induce the necessary percolation along the grain boundaries of PMMA in order to create an electron conductive pathway. This might result in lower transmittance in the visible region due to higher amounts of ITO particle addition.

**Figure 7:**
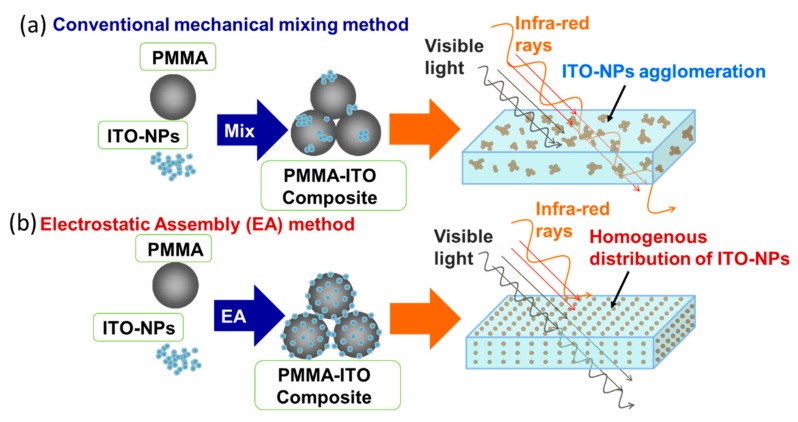
Illustration showing the comparison of the PMMA-ITO composite particles and pellets fabricated using (**a**) conventional mechanical mixing method and (**b**) electrostatic assembly method as well as its corresponding light transmission characteristics in the visible and IR region.

An illustration of the PMMA-ITO composite particles fabricated using a conventional mixing method and this novel electrostatic assembly method, as well as their visible light and IR rays transmittance, is shown in Figure 7. Due to the superior dispersion and homogeneity of ITO nanoparticles within the PMMA matrix obtained using the electrostatic assembly method compared to conventional mixing method, the transmittance properties in the visible region could be enhanced and at the same time achieving a better IR light shielding effect. Moreover, a more homogeneous distribution of ITO nanoparticles (without agglomeration) within the PMMA matrix would also improve the composite mechanical properties. The ITO nanoparticle agglomerates that exist at the interface of the PMMA particle would act as a crack initiation point where subsequent crack propagation through the microstructure would then lead to a fatal facture [16].

Besides pellet form, this finding on the homogenous formation of a PMMA-ITO composite via electrostatic assembly will also open up further possibilities for IR-cut PMMA-ITO composite fabrication in various dimensions such as sheet, fiber and rods that can be easily formed using extrusion or sheet forming methods.

## 4. Conclusions

A novel formation of PMMA-ITO composite particles was demonstrated using an electrostatic assembly method. The surface charge of the ITO nanoparticles was modified using PSS electrolyte, as well as by pH adjustment, and compared. Although both surface charge modifiers enabled homogenous distribution of ITO nanoparticles on the surface of PMMA, ITO nanoparticles that were surface-modified using pH adjustment exhibited better dispersion with no observation of agglomeration. The fabricated PMMA-ITO composite pellets exhibited good visible light transmittance of 80~90% (depending on the ITO nanoparticles surface charge modification) with a good and controllable IR light shielding effect. The IR light shielding effect of the PMMA-ITO composite can be controlled by adjusting the coverage percentage of ITO nanoparticles on PMMA. The use of the electrostatic adsorption method also allows better control of ITO nanoparticles distribution, which plays an important role in the plasmonic response towards generation of an electric field coupling and resonant splitting of plasmon excitations that are necessary for induction of selective IR light reflection. Prospective applications of this PMMA-ITO composite are IR active fillers for heat energy conservation as well as precision 3D holographic writing.
